# Reward-optimizing learning using stochastic release plasticity

**DOI:** 10.3389/fncir.2025.1618506

**Published:** 2025-08-14

**Authors:** Yuhao Sun, Wantong Liao, Jinhao Li, Xinche Zhang, Guan Wang, Zhiyuan Ma, Sen Song

**Affiliations:** ^1^Laboratory of Brain and Intelligence, Tsinghua University, Beijing, China; ^2^School of Biomedical Engineering, Tsinghua University, Beijing, China; ^3^School of Basic Medical Sciences, Tsinghua University, Beijing, China; ^4^Sapient Intelligence, Singapore, Singapore; ^5^College of Computer Science and Technology, Zhejiang University, Hangzhou, China

**Keywords:** synaptic plasticity, brain inspired computing, reinforcement learning, Spiking Neural Network, supervised learning

## Abstract

Synaptic plasticity underlies adaptive learning in neural systems, offering a biologically plausible framework for reward-driven learning. However, a question remains: how can plasticity rules achieve robustness and effectiveness comparable to error backpropagation? In this study, we introduce Reward-Optimized Stochastic Release Plasticity (RSRP), a learning framework where synaptic release is modeled as a parameterized distribution. Utilizing natural gradient estimation, we derive a synaptic plasticity learning rule that effectively adapts to maximize reward signals. Our approach achieves competitive performance and demonstrates stability in reinforcement learning, comparable to Proximal Policy Optimization (PPO), while attaining accuracy comparable with error backpropagation in digit classification. Additionally, we identify reward regularization as a key stabilizing mechanism and validate our method in biologically plausible networks. Our findings suggest that RSRP offers a robust and effective plasticity learning rule, especially in a discontinuous reinforcement learning paradigm, with potential implications for both artificial intelligence and experimental neuroscience.

## 1 Introduction

Synaptic plasticity serves as a fundamental mechanism for adaptive learning in neural systems (Bliss and Collingridge, 1993; Neves et al., 2008; Abbott and Nelson, 2000). A key characteristic of biological plasticity rules is their reliance on local information–such as pre- and postsynaptic neuronal activity–alongside global neuromodulatory signals. Among the most prominent of these rules is Hebbian plasticity (Hebb, 1949), which posits that synaptic strength is modulated by the correlated activity of connected neurons. This process can be further gated by global neural modulators, such as reward signals, leading to frameworks such as reward-modulated spike-timing-dependent plasticity (R-STDP) (Izhikevich, 2007). These models have demonstrated learning in biologically plausible paradigms and can be applied to tasks such as image classification (Diehl and Cook, 2015; Mozafari et al., 2019) and robotic locomotion control (Vasilaki et al., 2009).

The stochastic hedonic synaptic hypothesis posits an alternative learning mechanism, proposing that the learning process is primarily governed by reward-driven plasticity mediated through stochastic synaptic release (Minsky, 1954; Hinton, 1990; Seung, 2003). This phenomenon is modulated by the correlation between reward signals and probabilistic neurotransmitter release events. Crucially, it re-conceptualizes stochastic synaptic transmission not as biological noise but as a computational resource. By dynamically adjusting release probabilities in response to hedonistic feedback, synapses could stochastically explore and maximize expected rewards, thereby optimizing network functionality. This perspective challenges classical views of stochasticity in neural systems and highlights its potential role in reward-driven learning. Additionally, this hypothesis asserts that hedonistic synaptic learning is an approximation to the gradient of the average reward (Seung, 2003).

Hedonistic synaptic plasticity is potentially a more plausible learning mechanism in neural systems, compared to error backpropagation. Unlike backpropagation, which relies on continuous supervision signals and differentiable network architectures, reward-based plasticity operates without these constraints. However, several key questions remain unanswered, limiting the broader adoption of the stochastic hedonistic synaptic hypothesis. First, can a global reward signal alone suffice to achieve performance comparable to that of traditional methods, such as backpropagation? Second, how can such a learning process be scaled and regulated to accommodate larger and more complex neural networks for solving a non-trivial task?

To address these problems, we introduce a learning framework, Reward-Optimized Stochastic Release Plasticity (RSRP), inspired by the stochastic hedonistic synaptic hypothesis, to investigate its computational viability. In RSRP, synaptic release is modeled as a parameterized distribution, and learning occurs through optimizing these parameters to maximize global reward. To establish a theoretically grounded framework for reward-driven plasticity, we re-formulate the hypothesis using natural gradient descent, which is both effective and robust for optimizing a parameterized distribution (Wierstra et al., 2011; Wang et al., 2023). This yields a synaptic learning rule that operates in accordance with the hypothesis, ensuring mathematically principled adaptation to global reward signals.

Our approach achieves competitive performance in both reinforcement learning and classification tasks. Under comparable network architectures and parameter settings, RSRP demonstrates a comparable performance with Proximal Policy Optimization (PPO) (Schulman et al., 2017) in reinforcement learning tasks, and exhibits greater stability. In classification tasks, RSRP approaches backpropagation in accuracy while outperforming STDP-based plasticity rules.

Furthermore, we explore the learning process itself, describing how synaptic uncertainty evolves as the network learns and how it differs from gradient descent based approaches. We track the evolution of the probability distribution and find that the information entropy of the network decreases during the learning process, quantifying its information gain. The learning rule drives synapses from an uncertain state to a more certain state, a trend also observed in experiments (Zhou et al., 2024). Additionally, we identify that reward regularization is a crucial mechanism for stabilizing our learning rule, ensuring effective convergence.

Finally, we address the role of biological network architecture in learning. Traditional backpropagation-based networks often violate Dales Law and are not designed for balanced excitation-inhibition (EI) networks, limiting their applicability to biologically plausible learning (Lillicrap et al., 2020). In contrast, our approach demonstrates the ability to learn successfully in such environments. We further evaluate our method in reservoir networks, widely used networks for biologically plausible circuits, and find that our rule remains effective within these architectures.

In summary, we introduce a robust plasticity learning rule that achieves competitive performance while adhering to fundamental neural principles. Moreover, we highlight several key properties of our learning framework to achieve robustness and effectiveness, which could be further experimentally tested in biological systems, paving the way for future neurobiological validation.

## 2 Materials and methods

**Figure 1:**
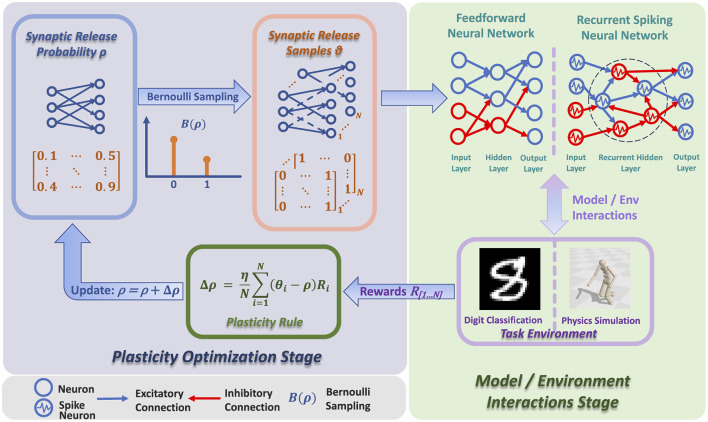
Architecture of RSRP, which consists of two stages: plasticity optimization stage and model/environment interaction stage. In the plasticity optimization stage, synaptic release probability is adjusted via Bernoulli sampling and plasticity rule. In the model/environment interaction stage, the neural network interacts with environments like physics simulation and digit classification, with rewards guiding the overall process.

As shown in Figure 1 and Algorithm 1, the architecture of RSRP contains two stages: the plasticity optimization stage and the model environment interaction stage. During the plasticity optimization stage, the synaptic release probability undergoes adjustment by applying the RSRP plasticity rule. In the model environment interaction stage, the neural network interacts with environments to acquire reward signals with synaptic release samples.

**Algorithm 1:**
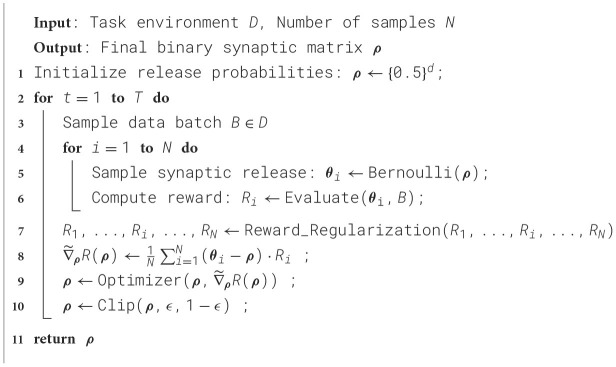
Reward-optimized stochastic release plasticity (RSRP).

### 2.1 Reward-optimized stochastic release plasticity

We extend the hedonistic plasticity learning rule framework (Seung, 2003) by introducing a parameterized Bernoulli distribution B(·) to model stochastic synaptic transmission. Specifically, we formulate the system's objective as an optimization problem where *J*(***ρ***) serves as the expected reward function quantifying system performance for a given release probability ***ρ***. We aim to determine the optimal release probability that maximizes expected reward:


(1) $$\begin{array}{l} \rho^{*}=\underset{\rho }{\text{argmax}}J(\rho )=\underset{\rho }{\text{argmax}}{𝔼}_{\theta \sim \text{B}(\rho )}[R(\theta )], \end{array}$$

where *R*(***θ***) corresponds to the reward signal received given a specific synaptic release sample ***θ***, which is a binary random variable representing either successful (***θ*** = 1) or failed (***θ*** = 0) synaptic transmission events. The learning rule governing the dynamic adjustment of ***ρ*** is formulated as


(2) $$\begin{array}{l} \Delta \rho =\frac{\eta }{N}\overset{N}{\underset{i=1}{\sum }}\left(\theta_{i}-\rho \right)R_{i} \end{array}$$

This equation specifies a learning mechanism that dynamically adjusts ***ρ*** through reward modulation. Specifically, the update Δ***ρ*** represents the adjustment direction and magnitude for synaptic efficiency optimization, where the learning rate parameter η controls the temporal scaling of updates, *R*_*i*_ and ***θ***_*i*_ are the reward signal and synaptic release samples in the *i*_*th*_ trial over *N* observed synaptic events, respectively.

To constrain the release probability **ρ** within the admissible domain [0, 1]^*K*^, we incorporated a projection operator with clipping threshold ϵ. The clipping approach is formally expressed as:


(3) $$\begin{array}{l} \rho^{\left(t+1\right)}=\text{clip}\left(\rho^{\left(t\right)}+\Delta \rho^{\left(t\right)},\;\epsilon ,\;1-\epsilon \right) \end{array}$$

### 2.2 Natural gradient estimation

While the standard gradient identifies the direction of steepest descent in Euclidean space, the natural gradient determines the steepest descent direction in the space of probability distributions, using the Kullback-Leibler (KL) divergence as a measure of distance. The natural gradient exhibits greater stability when optimizing over probability distributions (Wierstra et al., 2011). Synaptic release can be conceptualized as a form of probabilistic inference, thereby operating within a distributional space (Kappel et al., 2015). Building upon our framework, where the stochastic synaptic transmission is modeled as ***θ*** ~ B(***ρ***), the gradient of the expected reward function *J*(***ρ***) can be derived as follows:


(4) $$\begin{array}{l} \nabla_{\rho }J\left(\rho \right)={𝔼}_{\theta \sim B\left(\rho \right)}\left[\nabla_{\rho }\log P\left(\theta |\rho \right)R\left(\theta \right)\right] \end{array}$$

(5) $$\begin{array}{l} ={𝔼}_{\theta \sim B\left(\rho \right)}\left[\frac{\theta -\rho }{\rho \left(1-\rho \right)}R\left(\theta \right)\right] \end{array}$$

(6) $$\begin{array}{l} \approx \frac{1}{N}\overset{N}{\underset{i=1}{\sum }}\frac{\theta_{i}-\rho }{\rho \left(1-\rho \right)}R_{i} \end{array}$$

The equation shows that, unlike chain rule and backpropagation, the derivative of expected reward *J*(ρ) can be acquired without relying on an explicit calculation of gradient over a neural network. This technique aligns with a foundational concept in deep reinforcement learning, where the gradient of the expected reward is computed through the gradient of a probability distribution, thereby eliminating the need to directly differentiate the reward function. The key distinction in RSRP lies in how the probability distribution is defined: while deep reinforcement learning uses a neural network to parameterize the distribution, RSRP employs a straightforward Bernoulli distribution and treats the network solely as a component of the reward sampling mechanism.

To mitigate the instability commonly observed in standard gradient estimates (Wierstra et al., 2011), we instead employ natural gradient estimation:


(7) $$\begin{array}{l} {\tilde{\nabla }}_{\rho }J\left(\rho \right)=F^{-1}\nabla_{\rho }J\left(\rho \right) \end{array}$$

where $\tilde{\nabla }$ denotes natural gradient, and ***F*** denotes the Fisher information matrix. Given that the synaptic release process is assumed to be independent of each other neuron, the Fisher information matrix ***F*** takes a diagonal form:


(8) $$\begin{array}{l} F={𝔼}_{\theta \sim B\left(\rho \right)}\left[\nabla_{\rho }\log P\left(\theta |\rho \right)\nabla_{\rho }\log P{\left(\theta |\rho \right)}^{T}\right] \end{array}$$

(9) $$\begin{array}{l} =diag\left(\frac{1}{\rho \left(1-\rho \right)}\right) \end{array}$$

As a result, we derive a plasticity rule based on the natural gradient:


(10) $$\begin{array}{l} \Delta \rho =\eta {\tilde{\nabla }}_{\rho }J\left(\rho \right)=\eta F^{-1}\nabla_{\rho }J\left(\rho \right) \end{array}$$

(11) $$\begin{array}{l} \approx \frac{\eta }{N}\overset{N}{\underset{i=1}{\sum }}\left(\theta_{i}-\rho \right)R_{i} \end{array}$$

### 2.3 Relation to hedonistic synaptic plasticity

Seung (2003) proposed a learning rule that employs the sigmoid function σ to parameterize the synaptic release probability *p*. This approach utilizes an eligibility *e* to compute the gradient of the reward signal, incorporating a temporal summation trace mechanism, which might be similar to our summation over different synaptic samples. The learning rule can be summarized as:


(12) $$\begin{array}{l} p=\sigma \left(\rho \right) \end{array}$$

(13) $$\begin{array}{l} e=\theta -p \end{array}$$

(14) $$\begin{array}{l} \Delta q=\eta \underset{t}{\sum }e*R \end{array}$$

where ***θ*** denotes a synaptic release sample. Specifically, it employs a standard gradient approach, distinct from RSRP, to optimize the expected cumulative reward:


(15) $$\begin{array}{l} \nabla_{q}J\left(q\right)={𝔼}_{\theta \sim P\left(q\right)}\left[\nabla_{q}\log P\left(\theta |q\right)R\left(\theta \right)\right] \end{array}$$

(16) $$\begin{array}{l} ={𝔼}_{\theta \sim P\left(q\right)}\left[\sigma^{\prime }\left(q\right)\frac{\theta -p}{p\left(1-p\right)}R\left(\theta \right)\right] \end{array}$$

(17) $$\begin{array}{l} \approx \frac{1}{N}\overset{N}{\underset{i=1}{\sum }}\left(\theta_{i}-q\right)R_{i} \end{array}$$

Coincidentally, both update rules exhibit similar functional forms. However, two key differences can be identified: RSRP applies a natural gradient, whereas hedonistic plasticity employs a standard gradient; and RSRP updates the probability parameter directly, while hedonistic plasticity adjusts a parameter that indirectly controls the release probability through the sigmoid function.

To further enhance training stability in recurrent architectures, we sample the vesicle release event once per synapse at the beginning of each episode and hold it fixed across all time steps within that episode. This temporally shared randomness reduces the variance of learning signals, encourages temporal consistency in recurrent dynamics, and remains consistent with interpreting vesicle release reliability as a synapse-level trait that is modulated over behavioral episodes, rather than at every individual spike. While the model captures key computational principles, its biological fidelity could be further enhanced–an important direction for future research.

### 2.4 Regularization

#### 2.4.1 Centered rank based reward transform

To avoid local minima in the estimation process (Wierstra et al., 2011; Salimans et al., 2017), we employ a rank-based transformation over rewards. Instead of using the actual numerical values of individual rewards, we replace them with their ordinal rankings to construct the reward signal for the learning rule. Specifically, the transformed reward $R_{i}^{\prime }$ is computed as,


(18) $$\begin{array}{l} R_{i}^{\prime }=-\frac{1}{2}+\frac{1}{N}\underset{j}{\sum }\delta \left(R_{i}>R_{j}\right) \end{array}$$

where δ(·) denotes the indicator function, which equals 1 if *R*_*i*_ > *R*_*j*_ and 0 otherwise. Raw rewards, *R*, are converted to normalized, rank-ordered values that are subsequently centered at zero via the subtraction of $\frac{1}{2}$. Furthermore, this method of rank-based normalization finds a conceptual parallel in neuroscience. The functional diversity observed in midbrain dopamine neurons supports the concept of a distributional reward coding framework (Lowet et al., 2020). It is plausible that this neural mechanism serves a computational role analogous to the rank-based encoding proposed in our paradigm.

#### 2.4.2 Balanced input transformation

The input to a network may be unbalanced, exhibiting a nonzero mean or an asymmetric distribution, which can degrade the performance of a balanced network. Therefore, we expand the input into an EI-balanced form. Specifically, for any given input *x*, the input is transformed into:


(19) $$\begin{array}{l} x^{\prime }=concat\left(x,-x\right) \end{array}$$

By concatenating the input with its negated copy, we ensure that the resulting vector remains balanced. This mechanism mirrors biological sensory systems, where signals are processed in on-off cell pairs to maintain balance.

#### 2.4.3 Reward signal for classification

We employ three reward signals for the classification task: cross-entropy, accuracy, and a smoothed variant of recall, which we refer to as soft recall. In soft recall, the reward signal is computed using a smoothed function inspired by reciprocal rank. Specifically, for a given batch of data samples, the reward is defined as:


(20) $$\begin{array}{l} R=\frac{1}{TP+FP}\sum \frac{1}{{rank}_{i}} \end{array}$$

Where *rank*_*i*_ denotes that the position of the label *l*_*i*_ in the ranking of the network's classification outputs, in descending order. This formulation represents a modified approach to recall calculation, utilizing reciprocal rank to provide a smoothed estimation of true positives.

### 2.5 EI balanced networks

Excitatory-inhibitory (EI) balance is a fundamental property of neural networks. The output of an excitatory neuron is inherently positive, whereas the output of an inhibitory neuron is negative. Consequently, for a layer producing positive vectors–such as spike outputs in LIF neurons or ReLU activations in deep learning–its output can be transformed into an EI-balanced regime. This transformation is expressed as:


(21) $$\begin{array}{l} y=W@x^{\prime }\;x^{\prime }=concat\left(x_{0:N/2},-x_{N/2,N}\right) \end{array}$$

where both *x* and *W* are strictly positive. Notably, the positivity constraint on *W* can be naturally enforced within the RSRP.

### 2.6 Recurrent Spiking Neural Networks

For reinforcement learning, we employ a recurrent network composed of Leaky Integrate-and-Fire (LIF) neurons with exponential synapses. This network features a recurrent hidden layer that is directly connected to both the input and output layers, receiving environmental states as inputs and generating corresponding actions as outputs. The size of the hidden state in every RL task is 256. Each layer in the Recurrent Spiking Neural Network (RSNN) maintains excitatory-inhibitory (EI) balance. The input layer, output layer, and recurrent hidden layer are all trainable under the RSRP framework. Additionally, we implement a reservoir network with the same architecture, in which the recurrent hidden layer remains frozen, preserving a fixed set of random connections to provide a dynamic but unstructured computational regime.

### 2.7 Experimental setup

Our experiments mainly focused on two fields: Reinforcement Learning and Supervised Learning.

#### 2.7.1 Reinforcement learning (RL)

RSRP is evaluated on two RL benchmark tasks: the CartPole and Humanoid locomotion. These tasks represent complementary challenges spanning low-dimensional nonlinear instability and high-dimensional, contact-rich dynamics, offering a comprehensive assessment of RL capabilities across varying state-space complexities. Specifically, the CartPole task (2 DoF, simpler) and humanoid task (17 DoF, more complex) require policies to maximize the episodic return $R\left(\theta \right)={𝔼}_{\tau \sim \pi_{\theta }}\left[\overset{T}{\underset{t=0}{\sum }}r_{t}\right]$ through continuous torque optimization under distinct constraints. All models are trained and tested in the same version of the environment: the Gym (Brockman et al., 2016) environment for CartPole task, and Brax (Freeman et al., 2021) for Humanoid task. Each evaluation roll-out *R*_*i*_ corresponds to one full episode trajectory generated by policy parameters θ_*k*_: the CartPole task enforces strict actuator ranges for balance maintenance, while the humanoid imposes joint torque limits and fall-prevention termination conditions.

In our framework, the synaptic release sampling number refers to the number of samples used during synaptic updates, denoted as *N* in the update rule. In each RL task, *N* = 10, 240, ϵ = 0.001, and the learning rate is set at 0.15. We applied 100 training iterations in the CartPole task and 2,000 in the Humanoid task.

#### 2.7.2 Supervised learning

We further evaluate our learning rule on two benchmark supervised learning tasks: MNIST and CIFAR-10. The MNIST dataset consists of 60,000 training images of handwritten digits while CIFAR-10 comprises 50,000 training images across 10 object classes, both with 10,000 test images. To assess scalability across architectures, we employ both multilayer perceptrons (MLPs) and convolutional neural networks (CNNs). For MLPs, we use a single hidden layer with 64 units for MNIST and 128 units for CIFAR-10. The CNN architecture consists of two 3 × 3 convolutional layers with 32 and 64 channels, respectively, followed by a fully connected classification layer.

**Figure 2:**
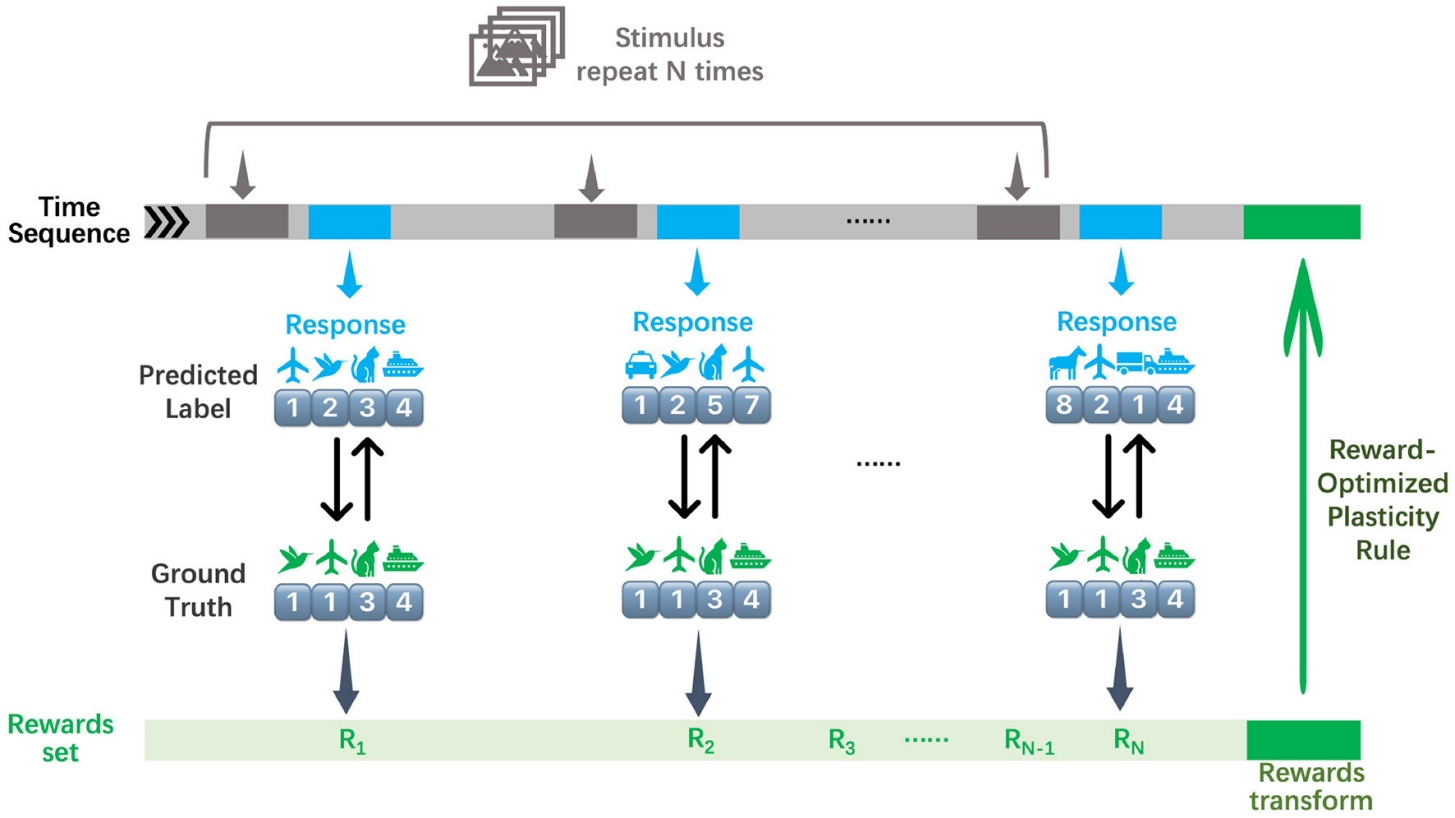
Illustration of the sparse reward training procedure of RSRP, where the networks are fed with *N* stimuli of an image batch and output predictions. After collecting all rewards from *N* trials, these rewards are transformed and guide the optimization of network parameters via the reward-optimized plasticity rule.

To align with the constraints of our proposed framework, we exclusively employ a sparse reward signal rather than conventional supervision. Specifically, instead of utilizing the standard cross-entropy loss, we define the reward as a binary logit-label hit signal, which assigns a positive outcome only when the models predicted classes are close to the ground-truth label. The design mirrors biologically plausible reinforcement paradigms, where discrete success/failure signals–rather than continuous error gradients–drive learning. By replacing dense error signals with a sparse reward structure (Figure 2), we emulate scenarios in which agents receive limited, intermittent feedback, thereby testing the robustness and efficiency of our rank-based learning mechanism under resource-constrained conditions.

For classification tasks, the data sampling size is defined as the number of input instances used to compute each reward signal per synaptic sample, as illustrated in Figure 2. In the baseline configuration, all synaptic samples within a training step utilize the same data batch. In contrast, subsequent studies adopt random data sampling for each forward trial to enhance variability and robustness. To achieve optimal performance, the number of synaptic release samples was set to *N* = 20, 000 for the MLP model and *N* = 8, 000 for the CNN model. While these values may exceed biologically plausible limits, we demonstrate that the RSRP framework remains capable of learning with significantly smaller, biologically realistic sampling sizes, albeit with a corresponding decline in task performance. Training was conducted over 5,000 steps (corresponding to 5 epochs) with a batch size of 64 for the MNIST dataset, and 5,000 steps (approximately 13 epochs) with a batch size of 128 for the CIFAR-10 dataset. Subsequent investigations focus on the MNIST-MLP64 configuration, employing 3,000 training steps to ensure computational efficiency while maintaining statistical reliability. All optimizations use simple updating rules (stochastic gradient descent, SGD) without momentum or weight decay mechanisms. Learning rates are tuned independently for each configuration to maximize performance.

Hyperparameters in our experiments for reproduction is listed in Supplementary material. Notably, RSRP exhibited stability in experiments, demonstrating no significant dependence on hyperparameter tuning. The only critical hyperparameter is the synaptic sampling size, which requires sufficiently large values to reduce optimization noise.

## 3 Results

### 3.1 Reinceforcement learning

#### 3.1.1 Comparative frameworks

Our experimental design systematically compares RSRP and its Reservoir computing (Jaeger, 2001; Jaeger and Haas, 2004) variant (RSRP-Reservoir) against three representative methods, Reward-modulated Spike-Timing-Dependent Plasticity (R-STDP), Evolutionary Strategy (ES) (Salimans et al., 2017), and Proximal Policy Optimization (PPO) (Schulman et al., 2017), spanning a range of optimization approaches, from biological plausibility to gradient-free optimization and mainstream RL methods. R-STDP-based Spiking Neural Networks (Haşegan et al., 2021) (SNNs) provide a neurophysiologically grounded baseline, testing the sufficiency of bio-inspired heuristics in complex tasks. ES-based R-SNN, which evolves network parameters through population-based sampling, probes the robustness of optimization when disentangled from gradient dynamics. Meanwhile, PPO with Long Short-Term Memory (LSTM) (Hochreiter and Schmidhuber, 1997) architectures represent the state-of-the-art gradient-based approach, establishing performance ceilings for backpropagation-driven learning. This systematic comparison–from strict biological mimicry to engineered gradient exploitation–demonstrates RSRP's unique ability to balance biological fidelity, training stability, and task efficiency, positioning it as a versatile solution for biological learning.

#### 3.1.2 Performance across learning horizons

**Figure 3:**
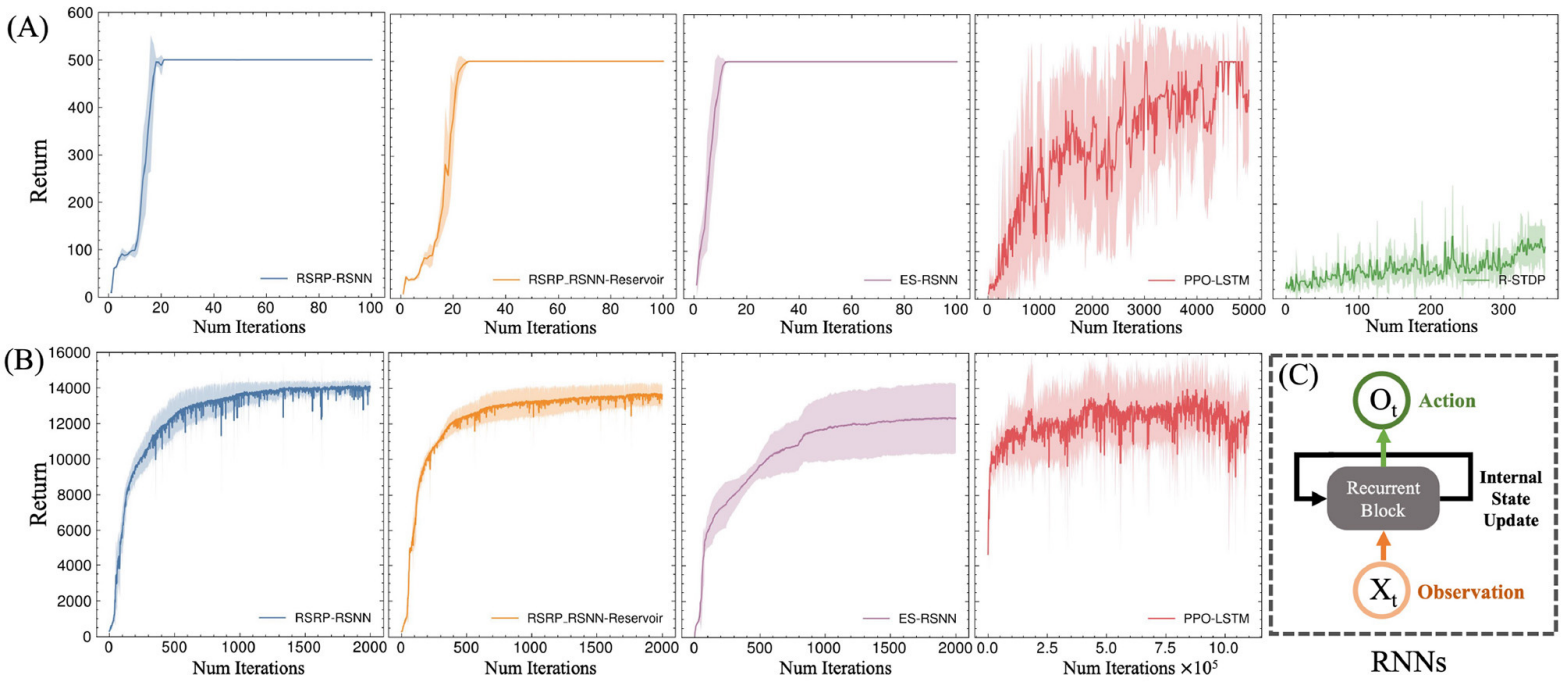
Evaluation of return during training on two typical locomotion reinforcement learning tasks: **(A)** CartPole task with 2 DoF and **(B)** Humanoid task with 17 DoF. **(C)** All the network are a variant of RNN. Five independent experimental trials are launched for each model. The plotted curves represent the arithmetic mean of the five replicate experiments, with the shaded regions indicating the range of standard deviation.

**Figure 4:**
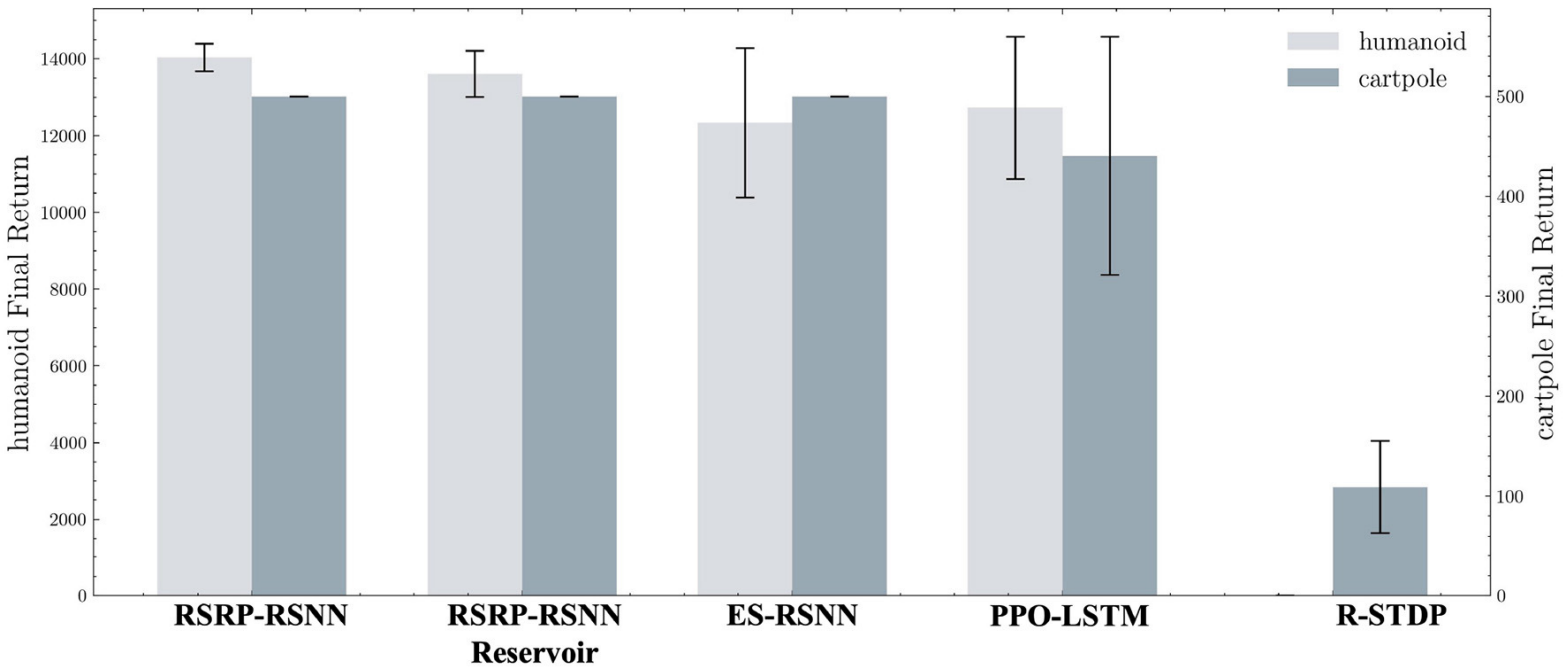
Average of final returns in trials of Humanoid and CartPole tasks. The left and right y-axes represent Humanoid final return and CartPole final return. Each bar is accompanied by error bars to visualize the data distribution.

Our methodology establishes robust performance benchmarks, as demonstrated by the learning trajectories (Figure 3) and final returns (Figure 4, Table 1). Researchers proved that reporting only the optimized performance highlights the best possible result from the algorithm, rather than its expected behavior in realistic settings. Across various fields, analyzing performance distributions is commonly recommended to offer a fuller picture of an algorithm's behavior (Jordan et al., 2020). Therefore, our methods focus more on the training curves and final rewards, instead of the peak performance during training. In the CartPole environment (Figure 3A), both RSRP-RSNN and its Reservoir variant match PPO-LSTM's maximum return (500), which is the maximum return of the environment. While our approach demonstrates rapid convergence, the learning trajectory reveals that R-STDP, a foundational plasticity optimization method, exhibits more gradual performance progression, stabilizing below a cumulative return of 300 across all training runs. In the more complex Humanoid environment (Figure 3B), our method demonstrates a more nuanced superiority: while PPO-LSTM occasionally generates high-return occasionally during training, our method consistently achieves higher final performance (14,033.97 vs 12,725.04). Notably, the Reservoir variant maintains 97% of base model performance with a frozen recurrent layer, consolidate the effectiveness of our plasticity framework on reservoir computing. This cross-task dominance confirms plasticity based approaches can rival backpropagation-based methods in a complex reinforcement learning scenario.

**Table 1:** Average of final returns in CartPole (2 DoF) and Humanoid (17 DoF), and the **bold** values indicate the best performance among all models.

**Method**	**Model**	**CartPole(2 DoF)↑**	**Humanoid(17 DoF) ↑**
R-STDP	SNN	109.00 ± 45.99	—
ES	R-SNN	**500.00** **±** **0.00**	12,331.52 ± 1,944.22
PPO	LSTM	440.36 ± 119.28	12,725.04 ± 1,852.03
RSRP	R-SNN-reservoir	**500.00** **±** **0.00**	13,606.16 ± 604.51
RSRP	R-SNN	**500.00** **±** **0.00**	**14,033.97** **±** **360.33**

#### 3.1.3 Robustness through synaptic stability

The learning curves' variance (Figure 3 shaded regions) and the variance of final returns (Figure 4) quantitatively validate our method's stability advantages over gradient-based counterparts. Where PPO-LSTM exhibits high and changing variance (±119.28 in CartPole; ±1,852.03 in Humanoid), our approach demonstrates stable variance control throughout training, achieving consistent performance in CartPole (500±0) and maintaining less than 2.6% relative Humanoid variance (360.33/14,033.97) versus PPO's 14.5% (1,852.03/12,725.04). Although PPO-LSTM exhibited transiently high returns during training, its overall performance was unstable, resulting in a lower final average performance relative to its peak performance. In contrast, RSRP demonstrated greater stability and consequently achieved a higher final average performance.

#### 3.1.4 Environment sampling efficiency between RSRP and PPO in humanoid task

**Figure 5:**
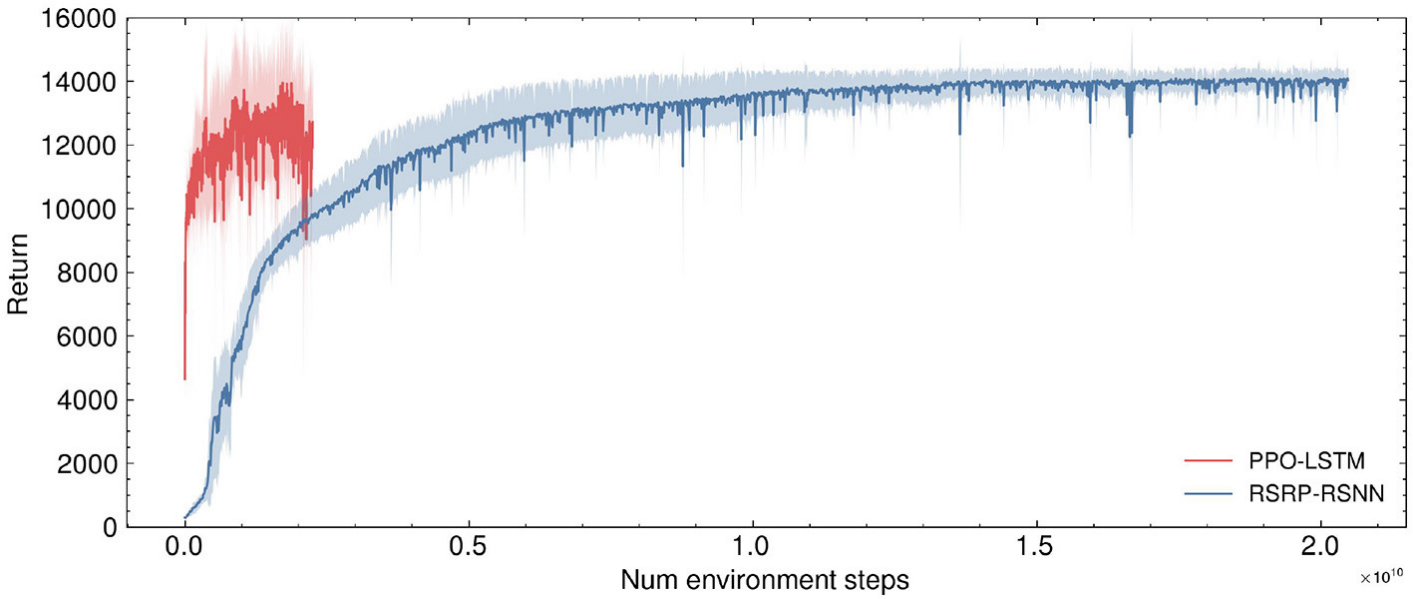
Comparison of environment sample efficiency during training for the humanoid task. A significant difference is observed in the environment sample efficiency between PPO and RSRP. RSRP and ES require the final return from the entirety of an episode for training. The number of environmental steps per episode varies with the “living time”, which in this task is less than 1,000. For standardization purposes, we consider each episode to consist of 1,000 environmental steps (though, in practice, our method typically uses even fewer steps). In contrast, PPO only requires 16 steps (specifically in this task) to perform optimization.

As shown in Figure 3B and Figure 5, the Humanoid task reveals a significant difference in environmental steps utilized between PPO and RSRP methodologies. In contrast to PPO, which optimizes policies using fragmented 16-step (specific to our experimental settings) trajectory segments, RSRP-based approaches rely on full-episode returns for gradient estimation, with episode lengths dynamically determined by the agent's survival duration (capped at 1,000 steps in this task). To ensure a fair comparison, we treat RSRP's episodic interactions as equivalent to 1,000 environmental steps per update cycle. However, empirical observations suggest that actual episode durations are typically much shorter. The results indicate that RSRP requires 8 × more total environment steps compared to PPO, however it requires × 550 fewer model parameter updates and demonstrating much more stability in learning. This operational distinction creates a efficiency trade-off: while PPO requires fewer environmental steps per update and achieves higher-frequency policy updates, it necessitates a significantly larger number of updates to complete training. In contrast, RSRP requires more environmental steps per update, leading to fewer but more stable updates.

### 3.2 Supervised learning

#### 3.2.1 Performance and stability

**Figure 6:**
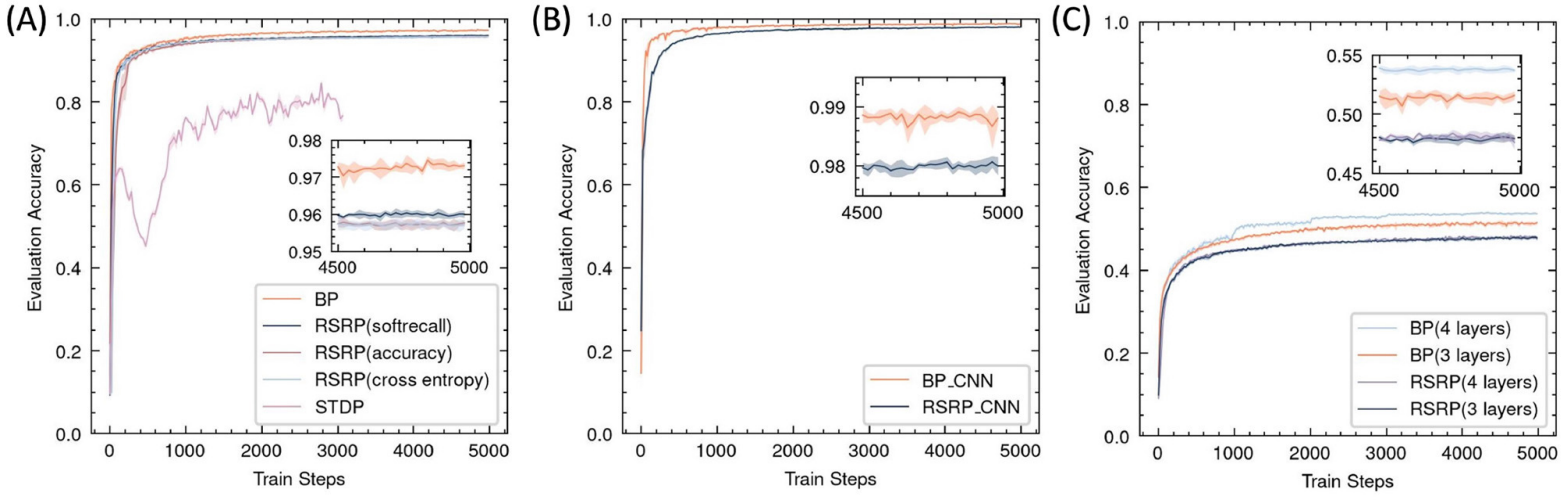
Performance of RSRP in supervised learning evaluated by accuracy of test set. Shaded regions denote standard deviation across five trials. **(A)** Training curves of a 3-layer MLP on MNIST, evaluating RSRP (0.96) with different reward functions (soft recall, accuracy, cross-entropy against BP (0.972) and STDP (0.85). **(B)** Performance of RSRP in training CNN on MNIST (0.98), compared to BP (0.988). **(C)** Performance on CIFAR-10 using deeper architectures (784-128-64-10), comparing between RSRP (0.48) and BP (0.54).

In the MNIST classification task, RSRP demonstrated comparable convergence performance and training stability to Backpropagation (BP) (Figure 6A). After RSRP training, the two layer fully connect networks stably achieved a test accuracy of 0.96 with consistent convergence, which was only 1 percentage points lower than that attained by BP. Notably, RSRP significantly outperformed Spike-Timing-Dependent Plasticity (STDP), a biologically plausible learning rule. While Diehl and Cook (2015) reported classification accuracy up to 95% using unsupervised STDP, our experimental replication demonstrated lower performance (85% maximum accuracy) with frequent divergence during optimization. The extension of the width to 1000 hidden neurons of the networks improved the performance up to 97.8%. Table 2 compares our results with other optimizing algorithms for the similar network architecture on MNIST.

**Table 2:** Comparison with similar feedforward network architecture on MNIST.

**Learning rule**	**Network structure**	**Accuracy↑ (%)**
STDP (Diehl and Cook, 2015)	784-6400 with EI	95
BP-STDP (Tavanaei and Maida, 2019)	784-100-10	94
BP-STDP (Tavanaei and Maida, 2019)	784-500-150-10	97.2
Equilibrium propagation (Scellier and Bengio, 2017)	784-500-10	97
Equilibrium propagation (Scellier and Bengio, 2017)	784-500-500-10	98
STBP (Wu et al., 2018)	784-800-10	98.9
RSRP (Ours)	784-64-10	96
RSRP (Ours)	784-1000-10	97.8

We also investigated the impact of different reward functions on RSRP optimization in supervised learning. The results indicated that simply using accuracy as a reward yielded similar performance to cross-entropy, though it introduced slight fluctuations in the early learning phase. Accuracy provides a reward only when the network produces a correct answer; however, it should also offer a partial reward for responses that are close to the correct answer, even if not entirely accurate. Thus, we introduced a smoothed version of the recall matrix, which succeeded in stabilizing gradient updates and accelerating convergence.

To further assess the scalability of RSRP in complex learning scenarios, we evaluated its efficacy across varying network architectures and task difficulties. As illustrated in Figure 6B, applying the convolution architecture in feedforward networks improved RSRPs performance, achieving a test accuracy of 0.98 (versus 0.96 for MLPs) with reduced parameter counts and higher training efficiency. On the more challenging CIFAR-10 benchmark (Figure 6C), RSRP maintained stable training dynamics to attain a test accuracy of 0.48 but exhibited a performance gap relative to BP(0.51), which was more pronounced than that in MNIST. Increasing network depth improved BP's performance but had negligible benefit on RSRP and introduced instability and divergence failures during training, highlighting a limitation of RSRP in deeper architectures. The result suggests that RSRP is capable of training deeper networks, although its performance on such architectures remains suboptimal.

In summary, RSRP demonstrated competent but inferior performance relative to BP in classical supervised learning. On one hand, unlike BP that relies on the cross-entropy-based gradient in supervised classification, RSRP employs a simpler reward-based optimization signal, which contains much less information. Although this may result in slightly lower performance, it offers significantly greater biological plausibility. On the other hand, RSRP's design is focused on modulating spike probability, while disregarding the synaptic strength adjustments, which is central to rules like STDP. Therefore, to unlock broader and better performance, RSRP should be viewed not as a standalone algorithm, but as a component that likely requires a complementary plasticity rule to work alongside it.

#### 3.2.2 Impact of sample size

**Figure 7:**
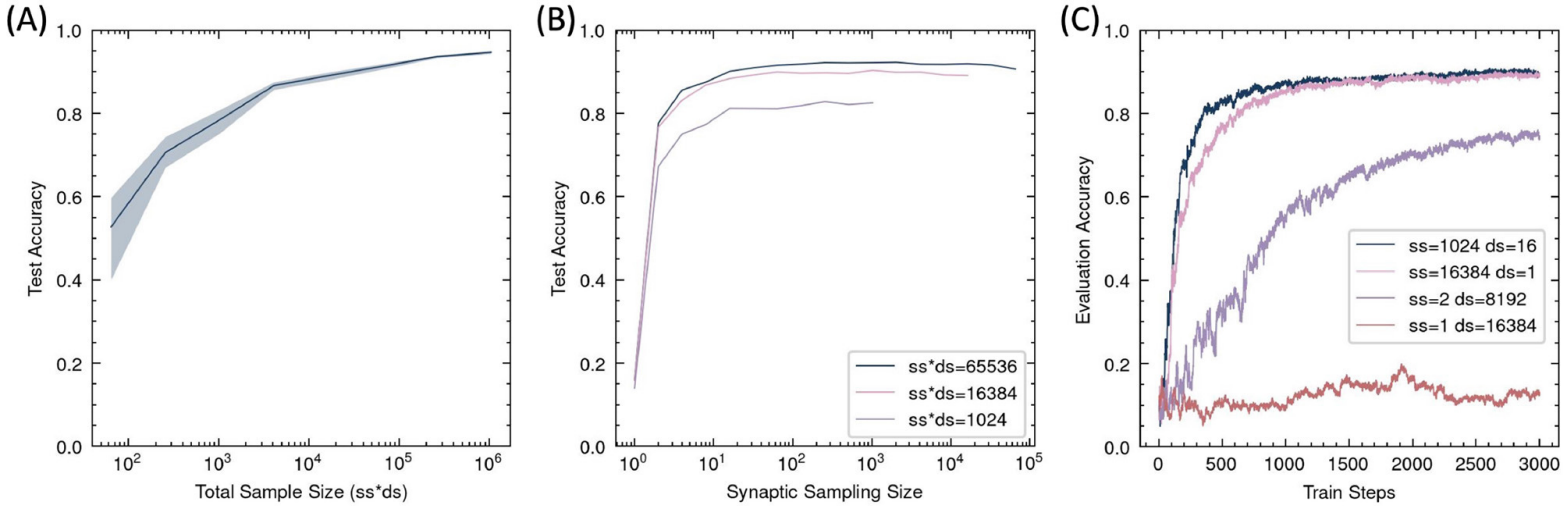
Task performance dependence on sampling number in RSRP. Data are sampled randomly for each sampling trial. **(A)** The Final test accuracy as a function of the total sample size per update step, given by the product of synaptic sampling size (ss) and data sampling size (ds). Shaded regions denote standard deviation across five different ss and ds. **(B)** Accuracy under fixed total sampling size (ss^*^ds = 1,024, 16,384, 65,536) with varying synaptic sampling sizes. **(C)** Training dynamics under extreme sampling configurations (ss=1 or ds=1).

Since RSRP estimates gradients stochastically as an expectation, it requires a number of samples to reduce variance. Thus, the precision of the gradient estimate and the optimization performance should primarily depend on the synaptic sampling size *N*, as indicated in Equation 2, while the batch size of the input data should have a relatively minor impact, as it mainly influences the precision of the reward *R*_*i*_. However, experimental results in the MNIST task (Figure 7) revealed that the final accuracy was determined by the product of synaptic sampling size and data sampling size, that is, the total number of forward trials processed per update step. This finding implies that if the total sample size remains constant, reducing the number of synaptic samplings while increasing the amount of input data preserves learning performance, unless it becomes too small. Without sampling in synapses, the algorithm fails to function. When synapse parameters are sampled only twice for gradient estimation, optimization occurs but remains suboptimal. At least a dozen synaptic events are required to achieve satisfactory learning outcomes.

#### 3.2.3 Learning progress of parameters

**Figure 8:**
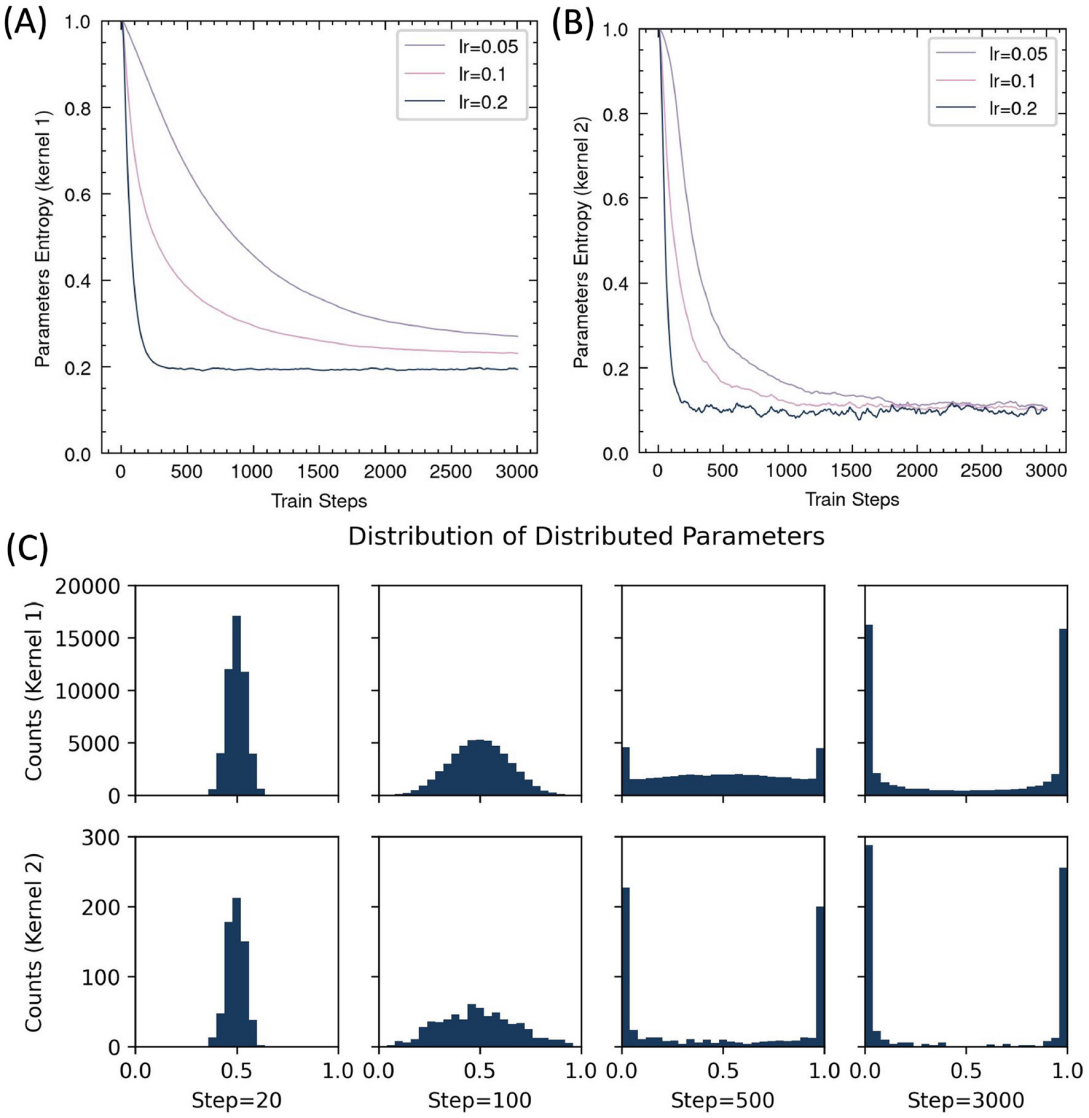
Evolution of distributed parameters during RSRP training. **(A, B)** Entropy dynamics of synaptic parameters across input-hidden layer (Kernel 1) **(A)** and hidden-output layer (Kernel 2) **(B)**. **(C)** Parameter distribution histograms at selected training steps.

The core principle of RSRP is reinterpreting synaptic plasticity as a parameterized stochastic distribution, it allows us to calculate the information entropy of the weights to quantify their transformation during learning. As training progressed, the entropy monotonically decreased (Figure 8), indicating that the network gradually accumulated more information. However, the entropy reduction in the input-to-hidden layer remained incomplete compared to the hidden-to-output layer, regardless of learning rate adjustments, suggesting insufficient learning in the first layer.

Moreover, we analyzed the evolution of distribution parameters during training. The distribution exhibits high symmetry, with parameters being gradually driven toward either zero or one by RSRP, while the overall mean remains centered at 0.5. This pattern suggested a structured consolidation of learned representations over time.

### 3.3 Regularizations

#### 3.3.1 Reward transformation

**Figure 9:**
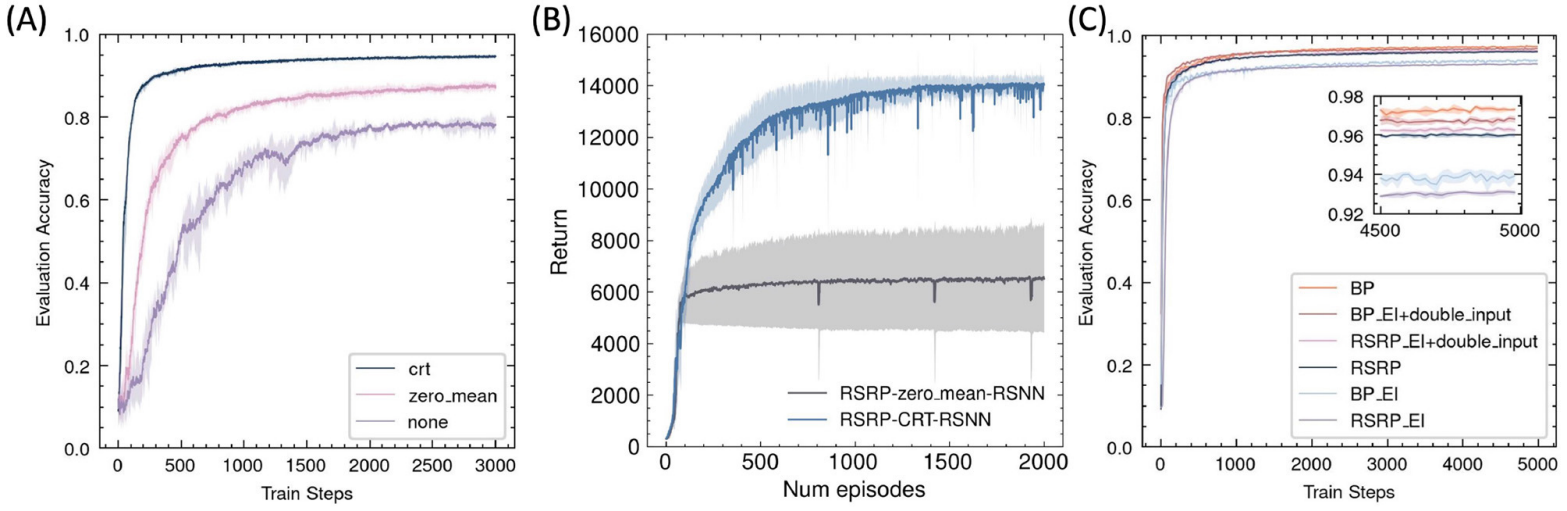
Effects of reward normalization and network architecture on RSRP performance. **(A)** Impact of reward transforms on MNIST classification, including centered rank transform (crt)(0.95), zero-mean normalization (0.88), and no transform (0.8). **(B)** Impact of reward transforms on humanoid task, including centered rank transform (CRT) (14,033), zero-mean normalization (6,534). **(C)** Performance of RSRP in training the excitation-inhibition balance networks compared with BP.

Without any reward regularization, the RSRP exhibited instability during training and achieved low final accuracy (Figures 9A, B). A simple and reasonable approach to maintaining network balance is to ensure a zero-mean reward by subtracting the history reward, which can be implemented by our brain. This adjustment improved optimization but remained inferior to the centered rank transformation. Thus, our results indicated that centered rank reward transform played a crucial role in the success of RSRP.

#### 3.3.2 Excitation-inhibition balance

By partitioning hidden-layer neurons into excitatory and inhibitory populations in equal proportion, we constructed an E-I balanced neural network that aligns better with biological plausibility than conventional ANNs. We observed that RSRP and BP performed similarly in optimizing this network, but both exhibited degraded performance compared to their performance in a standard ANN (Figure 9C). To maintain the balance of the input layer, we augmented the input data with a mirrored counterpart. With the modification, RSRP achieved performance levels that slightly outperformed those of the original ANNs, while BP did not exhibit complete recovery. These findings suggest that with appropriate input regularization, RSRP is capable of learning in an E-I balanced neural network.

#### 3.3.3 Other regularizations in BP

To enhance the training performance of RSRP, we investigated several regularization strategies commonly employed in BP. Weight decay is widely used in BP to mitigate overfitting in traditional deep learning. Here, we attempted to apply weight decay to RSRP by constraining the distributed parameters toward 0.5, but this approach yielded no performance improvement.

We also examined the impact of different optimizers, including momentum and Adam, and found no significant differences in RSRPs performance compared to vanilla SGD in most experimental configurations. However, in the optimization of a four-layer MLP trained on CIFAR-10, which was more unstable than a three-layer MLP, incorporating momentum effectively reduced the occurrence of divergence failures. These findings suggest that future work should focus on developing RSRP-specific optimization techniques to reduce noise within gradient decent.

## 4 Discussion

The diversity of learning mechanisms across brain regions suggests that neural circuits may employ specialized learning rules tailored to their functional roles. Hebbian plasticity is widely observed in the nervous system, and its mathematical models are useful for associative and unsupervised learning. Error backpropagation, another prominent learning mechanism, is well tested in supervised learning. However, it remains questionable whether the entire brain utilizes such a learning rule, given that it requires precise error signal backpropagation. Our findings propose a complementary framework RSRP: learning through global reward and synaptic plasticity. Nevertheless, we demonstrated that RSRP does not perform optimally across all tasks. This could be a prediction that learning in the brain could involve a combination of different learning rules, where distinct regions utilize specialized mechanisms suited to their respective functions. Notably, our experiments demonstrated that RSRP performs particularly well in motor control tasks. Reservoir networks exhibit competitive performance in these tasks, although they show a slight performance decay compared to trainable recurrent networks, which offer greater adaptability. This performance gap highlights the potential for further investigating the modification of statistical properties and their impact on motor control.

While learning rules with imprecise gradients, like RSRP, may not be optimal for training deep networks from scratch, we hypothesize they are highly effective for fine-tuning large models such as Transformers. Our central premise is that the performance of algorithms like RSRP is significantly enhanced when operating within a neural network's linear regime. We find empirical support for this in our experiments with reservoir computing, where RSRP achieves competitive performance by training an inherently linear input and readout layer. This principle is not limited to explicitly linear models: Neural Tangent Kernel (NTK) theory suggests that even complex, deep networks can operate in an effectively linear manner from the perspective of parameter updates (Jacot et al., 2018; Lee et al., 2019). This insight is particularly relevant for fine-tuning large, pre-trained models, where small parameter adjustments approximate linear dynamics. Therefore, while RSRP may be sub-optimal for end-to-end training, it is a promising candidate for the fine-tuning stage, which operates within this effective linear regime.

Notably, RSRP contrasts with canonical STDP, which operates through unsupervised, correlation-based updates dependent on presynaptic and postsynaptic activity. While STDP excels at refining temporal representations, its capacity to integrate explicit supervisory or reward-driven signals remains theoretically underexplored. Our results address this gap by demonstrating that reward-contingent synaptic modifications can drive task-specific learning without relying on precise spike timing. Distinct synaptic plasticity rules have been observed to operate across dendritic compartments during learning (Wright et al., 2025), suggesting that RSRP may plausibly function in parallel with other learning mechanisms. Crucially, RSRP posits that synaptic efficacy updates are gated by reward signals rather than solely by presynaptic release events, a distinction with testable and significant neurobiological implications. If validated experimentally, the identification of reward-triggered presynaptic probability modulation *in vivo*—potentially mediated by dopamine-dependent vesicular priming or long-range glutamatergic modulators such as mGluRs and NMDARs–would provide compelling evidence for our framework, particularly in supporting their role in inducing local dendritic depolarization (Sherman and Guillery, 1998).

Additionally, a key component of RSRP involves reward regularization, particularly rank-based regularization and the utilization of natural gradient. Biological systems often operate in noisy and variable environments, and ranking could be a strategy to enhance survival in such conditions. It may offer a framework for neuronal coding schemes within the brain, as suggested by previous studies on rank coding (Portelli et al., 2016). For instance, it could help explain the role of lateral inhibition in cortical neurons, which may serve to encode rank-based information. Such findings could also help elucidate the neuronal coding mechanisms underlying reward distribution, thereby contributing to more stable learning dynamics driven by reinforcement signals (Lowet et al., 2020).

Future studies could investigate whether RSRP-like dynamics are enriched in circuits associated with reinforcement learning, such as cortico-striatal pathways, and how they interact with Hebbian or STDP-driven processes. Such investigations would clarify how distinct learning rules are anatomically partitioned or dynamically recruited to support adaptive behavior.

## Data Availability

The original contributions presented in the study are included in the article/Supplementary material, further inquiries can be directed to the corresponding author.
