# Supplementary material for: Reward-optimizing learning using stochastic release plasticity
Source: Front Neural Circuits. 2025 Aug 14;19:1618506. doi: 10.3389/fncir.2025.1618506 (PMC12390965; doi:10.3389/fncir.2025.1618506)
Supplement: Supplementary file 1 [file Data_Sheet_1.pdf]

Supplementary Material

1 SOURCE CODE

Our supplementary data, including datasets access and implementation code, are publicly accessible via our GitHub repository at `https://github.com/Brainmatrix-songlab/RSRP`.

2 HYPERPARAMETERS

The appendix presents three tables detailing our hyperparameter configurations for the Cartpole, Humanoid, MNIST, and CIFAR-10 tasks.

For the Cartpole and Humanoid reinforcement learning tasks, we employed a synaptic sampling size of 10,240, which provides sufficient accuracy for estimation. The Cartpole task, being relatively simpler, requires only 100 training steps (converging in approximately 15 steps on average), while the RSRP algorithm requires 2,000 steps to converge on the Humanoid task.

Regarding the image classification tasks, we evaluated various optimizers and found minimal performance variation, ultimately selecting SGD. Through grid search across different hyperparameters (including learning rates ranging from 0.1 to 10), we observed that while all configurations eventually converged, they showed only marginal differences in final accuracy. The tables display the optimal parameter combinations we identified(* indicates networks with frozen hidden layers).

Table S1. Hyperparameter Configurations for Cartpole

**Hyperparameter Name**	**Value**
Network type	RSNN
Total Steps	100
Synaptic Sampling Size	10240
Learning Rate	0.15
Weight decay	0
Clip Epsilon	1e-3
Initialization Parameters	0.5

(a)RSRP-RSNN

**Hyperparameter Name**	**Value**
Network type	RSNN*
Total Steps	100
Synaptic Sampling Size	10240
Learning Rate	0.15
Weight decay	0
Clip Epsilon	1e-3
Initialization Parameters	0.5

(b)RSRP-RSNN-Resevior

**Hyperparameter Name**	**Value**
Network type	RSNN
Total Steps	100
Synaptic Sampling Size	10240
Learning Rate	0.01
Weight decay	0
Clip Epsilon	1e-3
Initialization Parameters	0.5

(c)ES-RSNN

**Hyperparameter Name**	**Value**
Network type	LSTM
Total Steps	5000
Reward Scale	0.005
Learning Rate	0.0003
Clip Epsilon	0.2
Gamma	0.98

(d)PPO-LSTM

Table S2. Hyperparameter Configurations for Humanoid

**Hyperparameter Name**	**Value**
Network type	RSNN
Total Steps	2000
Synaptic Sampling Size	10240
Learning Rate	0.15
Weight decay	0
Clip Epsilon	1e-3
Initialization Parameters	0.5

(a)RSRP-RSNN

**Hyperparameter Name**	**Value**
Network type	RSNN*
Total Steps	2000
Synaptic Sampling Size	10240
Learning Rate	0.15
Weight decay	0
Clip Epsilon	1e-3
Initialization Parameters	0.5

(b)RSRP-RSNN-Resevior

**Hyperparameter Name**	**Value**
Network type	RSNN
Total Steps	2000
Synaptic Sampling Size	10240
Learning Rate	0.01
Weight decay	0
Clip Epsilon	1e-3
Initialization Parameters	0.5

(c)ES-RSNN

**Hyperparameter Name**	**Value**
Network type	LSTM
Total Steps	1100000
Reward Scale	0.005
Learning Rate	0.0003
Clip Epsilon	0.2
Gamma	0.99

(d)PPO-LSTM

Table S3. Hyperparameter Configurations on image classification

**Hyperparameter Name**	**Value**
Network Size	64
Synaptic Sampling Size	20480
Data Sampling Size	64
Total step	5000
Optimizer	SGD
Learning Rate	4
Reward	Soft recall
Reward Transform	crt
Crt Scale	reward std=1
Clip Epsilon	1e-3
Initialization Parameters	0.5

(a)RSRP on MNIST(MLP)

**Hyperparameter Name**	**Value**
Network Size	128
Synaptic Sampling Size	8192
Data Sampling Size	64
Total step	5000
Optimizer	SGD
Learning Rate	6
Reward	Soft recall
Reward Transform	crt
Crt Scale	reward std=1
Clip Epsilon	1e-3
Initialization Parameters	0.5

(b)RSRP on MNIST(CNN)

**Hyperparameter Name**	**Value**
Network Size	32-64(3*3)
Synaptic Sampling Size	16384
Data Sampling Size	128
Total step	5000
Optimizer	SGD
Learning Rate	4
Reward	Soft recall
Reward Transform	crt
Crt Scale	reward std=1
Clip Epsilon	1e-3
Initialization Parameters	0.5

(c)RSRP on CIFAR-10(MLP)

3 SPIKE ACTIVITY

We analyzed the spiking dynamics of the Recurrent SNN to determine if it exhibits an irregular spiking paradigm. The network was analyzed after being trained on a humanoid task using the RSRP under reinforcement learning paradigm. For our experiments, the network was tuned to an optimal average recurrent strength, which is potentially the most representative state of the reservoir. As illustrated in Figure S1, the resulting spike train exhibits both rhythmic and irregular patterns. A temporal independence test on the 256 neurons showed that the spike trains of 148 neurons can be considered temporally independent, confirming a key property of irregular spiking. The rhythmic component is a direct result of the task, as the network generates a cyclical pattern to control a humanoid's running gait.

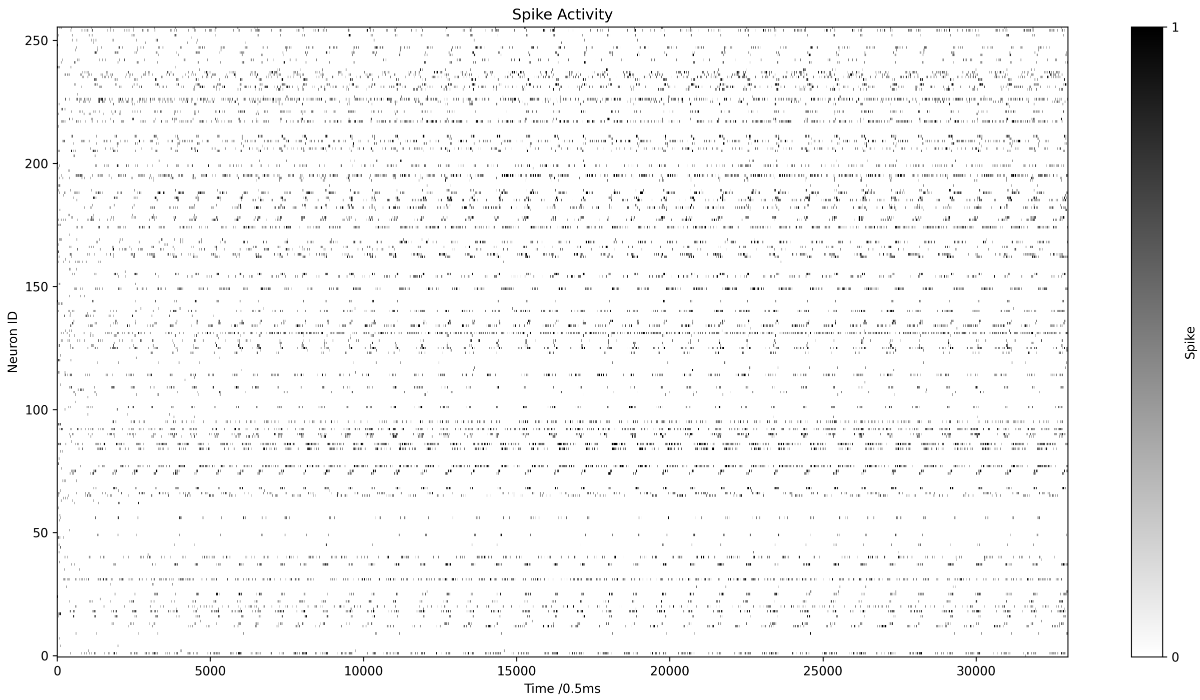

Figure S1: Spiking pattern of the recurrent spiking network after training on Humanoid

4 COMPARISON OF GRADIENT AND NATURAL GRADIENT

Although a formal proof of convergence is currently not available, we conducted a simulation study to illustrate the difference in convergence behavior between RSRP and its standard gradient counterpart, as shown in the figure below. In this experiment, we employed an intuitive and simple setting where rewards are issued contingent on synaptic release events. In addition, to simulate more realistic conditions, we introduced stochasticity in the reward signal: with a certain probability—referred to as the noise ratio—a reward is omitted despite a release event, and vice versa. Despite the noise, an ideal behavior under such paradigm is to always emit a spike, i.e, $\rho = 1$, for a maximum expected reward.

In the idealized scenario of a noise-free reward signal (i.e., noise ratio = 0), both the standard gradient and the natural gradient methods achieve convergence, as expected. In contrast, when rewards are noisy, the natural gradient method remains robust, whereas the standard gradient method fails to converge. This

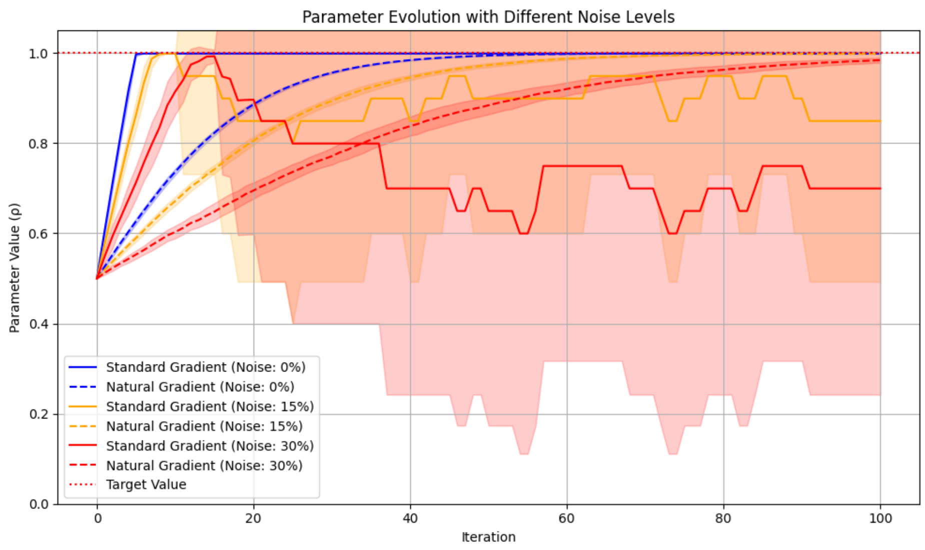

Figure S2: Comparison of convergence of parameter learned using standard gradient and natural gradient. Shaded area is variance across different trials.

observation underscores the robustness of the natural gradient in the context of noisy reward signals and highlights its potential advantage in scenarios where convergence under uncertainty is critical.
